# Abstracts from the Reports of Medical Officers of Health

**Published:** 1907-08-17

**Authors:** 


### ABSTRACTS FROM THE REPORTS OF MEDICAL OFFICERS OF HEALTH.

### THE PREVENTION OF CONSUMPTION.

Dealing with this important matter, Dr. T. Orme Dudfield, Medical Officer for the Royal Borough of Kensington, refers to the part played by compulsory notification and sanatorium treatment.

"I adhere to the opinion," he remarks, "that compulsory notification is indispensable to success in the administrative control of phthisis, a disease which, year by year, is the cause of more deaths than all the scheduled notifiable diseases combined. . . . . As regards London, however, it has regretfully to be admitted that the Local Authorities are not in a position and are not ready to deal effectively even with the cases voluntarily notified to them, practically no provision, outside the Poor-law Infirmaries, having been made for the isolation and treatment of sufferers. . . . . The economic importance of pulmonary phthisis does not yet appear to be adequately appreciated; and in England no effective steps are likely to be taken for its control and treatment until it is realised that not only thousands of lives but also millions of pounds sterling, would be saved every year, could consumption be relegated to the class of extinct diseases."

With regard to sanatorium treatment, Dr. Dudfield considers that the doubts as to the effectiveness of such treatment are unwarranted by results. "Such doubt may have arisen from observation of the poor, or apparently bad, results of treatment, in such institutions of unsuitable cases. But those who raise doubt generally fail to notice that for multitudes of poor sufferers there is little or no hope unless they can be removed from the bad surroundings of homes where they cannot be treated with advantage, and in which they are liable to spread disease amongst their families."

### THE RESULTS AT BRIGHTON.

In this connection Dr. Newsholme, Medical Officer of Health for Brighton, gives an interesting account in his annual report of the system of voluntary notification adopted in that town, and of the treatment and training of patients in the Isolation Hospital. From it we learn that municipal patients are usually admitted for a month each, and that no charge is made. From 1898 the Borough Laboratory has undertaken, free of charge, the examination of sputum sent by doctors from Brighton patients. Only selected cases, and such as promise a reasonable hope for cure are admitted. The system of notification has worked admirably.

"So far from patients objecting to notification, a not infrequent difficulty has been that they persist in applying for sanatorium treatment apart from the advice and certificate of a private doctor, upon which we always insist."

### MILK DEPOTS.

Dr. McCluny, Medical Officer of Health for Hampstead, gives in his report a brief but interesting dissertation on Municipal Milk Depots.

"The Infants' Milk Depot is not a milk shop. . . . . Its work is far from being restricted to the distribution of specially-prepared milk. The milk is only supplied to those mothers who can give good reasons for discontinuing to nurse the baby; and by advising applicants for the milk it is possible, as I can testify from my own experience, to persuade many mothers to continue nursing who would otherwise have prematurely weaned their babies. The milk depot . . . . is really a valuable educational influence. It does things that a milk company does not do. . . . . It must be borne in mind that many of the children who are brought to the depot have not been, and but for the depot would not be, fed upon cow's milk at all. Everyone who has worked among the poor knows that large numbers of hand-fed babies are not fed upon cow's milk but upon worthless patent foods and cheap brands of condensed milk."

In the same report Dr. McCluny admirably summarises the action taken by municipalities in their attempts to reduce the waste of infant life which persists despite the vast improvement otherwise noticeable in the vital statistics of the country.

"Although unwholesome surroundings play an important part in the causation of infantile mortality, a much more important factor is defective 'mothering.' Whether any given infant will survive to attain its first birthday depends above all things on the care and attention that is bestowed upon it. In the poorer districts infants do not receive proper care, not, however, because the mother is careless, but because she does not possess the necessary knowledge of infant life or the necessary facilities for infant management. . . . . As a matter of fact, all over the country the public health work of the municipality has very definitely been extended so as to include not only the hygiene of the environment, but also the hygiene of the person, and the education of mothers in the feeding and general care of infants has become part of the ordinary routine work of the municipal health department."

### INFANTILE MORTALITY.

An inquiry by Dr. William Butler, Medical Officer of Health of Willesden, into the relation of infantile mortality and the methods of rearing children, is the subject of some interesting tables, diagrams, and comments in his annual report.

"Unquestionably the most important fact bearing upon the prospects of survival of the infant is the manner of feeding. Out of 331 infants investigated dying within the year, no fewer than 60 per cent. were solely hand fed, while of 1,522 surviving to the end of the year under 12 per cent. were so fed.

"This is extremely significant, for it shows that, quite apart from the direct injury to the baby resulting from the deprivation of its natural food, these babies succumb in greater degree to all diseases by which they are attacked. In other words, artificially fed infants have a lowered vitality even when they survive. But when we consider that 88 per cent. of the children dying from zymotic diarrhœa are exclusively hand-fed, and that only 4 per cent. were alleged to be solely breast-fed, that 78 per cent. of those dying from tuberculosis, 66 per cent. of those dying from marasmus, and 63 per cent. of those dying from enteritis were exclusively hand-fed, it is impossible to avoid the conclusion that in this fact alone we have the chief factor in the production of these diseases.

"In respect of improper food, improper feeding bottles, and irregularity of feeding, infants dying within the year are much worse than those surviving. Among those surviving, that class is best in which all the others of the family of four or more survive, while the worst occur in the group in which three or more of the family have died. First-born infants are taken out of doors more than second, second more than third, and third more than fourth in the group where all survive; while the greater the number of children a mother has lost the less frequently is the last child taken out. Whether the mother is attended by a doctor or a midwife at her confinement appears to have no influence upon the prospects of the infant's surviving, but it is interesting to note that the proportion of mothers attended by midwives increases with the number of children they have previously borne. It is also interesting to note that the more children a mother has lost the more ready is she to promise to improve her methods in the care of the infant, and that the mothers of firstborn children are the least disposed to amend their ways or, at any rate, to admit that they will.

"Amendment of vital conditions, especially as regards infant mortality, is very much a question of improving the mothers. This is the greatest and most pressing sanitary problem of the day. The further improvement, not of drainage, nor housing, nor water supply, nor even of all that is comprehended under sanitary engineering is of first importance in a modern town. A proper perspective of the factors which chiefly under modern conditions determine disease and mortality cannot fail to give prominence, nay, pre-eminence, to the influence of ignorance and inaptitude. Even more important than the elementary education of girls is the secondary training of young women in the first principles and practice of domestic hygiene and in the management of the home and of the young family."

